# Excitatory Repetitive Transcranial Magnetic Stimulation Induces Contralesional Cortico-Cerebellar Pathways After Acute Ischemic Stroke: A Preliminary DTI Study

**DOI:** 10.3389/fnbeh.2018.00160

**Published:** 2018-07-27

**Authors:** Jing Li, Zhentao Zuo, Xuewei Zhang, Xiali Shao, Jie Lu, Rong Xue, Yong Fan, Yuzhou Guan, Weihong Zhang

**Affiliations:** ^1^Department of Radiology, Peking Union Medical College Hospital, Peking Union Medical College & Chinese Academy of Medical Sciences, Beijing, China; ^2^State Key Laboratory of Brain and Cognitive Science, Beijing MR Center for Brain Research, Institute of Biophysics, Chinese Academy of Sciences, Beijing, China; ^3^Department of Interventional Radiology, China Meitan General Hospital, Beijing, China; ^4^Department of Radiology, School of Medicine, University of Pennsylvania, Philadelphia, PA, United States; ^5^Department of Neurology, Peking Union Medical College Hospital, Peking Union Medical College & Chinese Academy of Medical Sciences, Beijing, China

**Keywords:** acute ischemic stroke, repetitive transcranial stimulation (rTMS), diffusion tensor imaging (DTI), fractional anisotropy (FA), stoke rehabilitation

## Abstract

**Background:** Repetitive transcranial magnetic stimulation (rTMS) is proved to be effective in facilitating stroke recovery. However, its therapeutic mechanism remains unclear. The present study aimed to investigate changes in white matter fractional anisotropy (FA) after excitatory rTMS to better understand its role in motor rehabilitation.

**Materials and Methods:** Acute stroke patients with unilateral subcortical infarction in the middle cerebral artery territory were recruited. The patients were randomly divided into an rTMS treatment group and a sham group. The treatment group received a 10-day 5 HZ rTMS applied over the ipsilesional primary motor area beginning at about 4 days after stroke onset. The sham group received sham rTMS. Diffusion tensor imaging (DTI) data were collected in every patient before and after the rTMS or sham rTMS. Voxel-based analysis was used to study the difference in FA between the two groups. The trial of this article has been registered on the *ClinicalTrials.gov* and the identifier is NCT03163758.

**Results:** Before the rTMS, there is no significant difference in FA between the two groups. Differently, after the treatment, the rTMS group showed increased FA in the contralesional corticospinal tract, the pontine crossing tract, the middle cerebellar peduncle, the contralesional superior cerebellar peduncle, the contralesional medial lemniscus, and the ipsilesional inferior cerebellar peduncle. These fasciculi comprise the cortex-pontine-cerebellum-cortex loop. Increased FA was also found in the body of corpus callosum and the contralesional cingulum of the treatment group compared with the sham.

**Conclusion:** The greater connectivity of contralesional cortico-cerebellar loop and the strengthening of interhemispheric connection may reflect contralesional compensation facilitated by the excitatory rTMS, which gives us a clue to understand the therapeutic mechanism of rTMS.

## Introduction

Stroke is a major cause of long-term disability (Writing Group Members Mozaffarian et al., 2016). Motor dysfunction leads to activity limitations of stroke survivors, which impairs their quality of life (Skolarus et al., 2014). Many interventions have been used to improve motor function after stroke, for instance, drug therapy, muscle strengthening exercises, motor learning, and so on. However, study indicated that after completed rehabilitation, more than 50% stroke patients were still mildly to severely disabled (Jørgensen et al., 1995). Therefore, potential treatment to promote motor recovery after stroke should be explored.

Repetitive transcranial magnetic stimulation (rTMS) is applying a train of TMS pulses of the same intensity to a certain brain area at a given frequency. It could modulate cortical excitability. High-frequency (≥5 HZ) rTMS tends to induce long-term potentiation of synaptic activity and low-frequency (<1 HZ) is more likely to have the opposite effect (Bliss and Cooke, 2011). Acting as a neurostimulator, rTMS is being increasingly used in treating neurological disorders. Several studies have proved its effectiveness in facilitating motor recovery after stroke (Khedr et al., 2005; Nowak et al., 2009; Yoon et al., 2011; Hosomi et al., 2016). Our former longitudinal study also indicated that the treatment effect could last for 1 month after onset (Guan et al., 2017). However, the therapeutic mechanism of rTMS remains poorly understood. Results from animal study speculated that high-frequency rTMS improved functional recovery possibly by enhancing neurogenesis and activating brain-derived neurotrophic factor (BDNF)/tropomyosin-related kinase B (TrkB) signaling pathway (Luo et al., 2017). Positron emission tomography revealed cerebral blood flow changes after rTMS treatment on stroke patients (Weiduschat et al., 2011). Using functional magnetic resonance imaging (fMRI), several studies detected increased interhemispheric functional connectivity after rTMS (Li et al., 2016; Mitaki et al., 2016). Combining rTMS and functional neuroimaging techniques enables us to explore brain changes after rTMS from metabolic and functional aspects. But this is not enough. To better understand the pathophysiological mechanism of rTMS, it is essential to figure out whether these brain changes are merely functional or practically structural.

Diffusion tensor imaging (DTI), as a non-invasive approach to display and analyze white matter integrity *in vivo*, can provide us with information on this. It measures the diffusion effects of water molecules. Water diffuses more quickly along with white matters and more slowly perpendicular to the fibers, resulting in anisotropic diffusion (Mukherjee et al., 2008). Fractional anisotropy (FA) is a diffusion metric that characterize anisotropic diffusion. It quantifies motion of water molecules which preferentially along the axis of axonal pathways. As motion becomes more coherent and aligned, FA values approach 1.0 (George et al., 2014). FA could reflect integrity of brain fibers, increases in FA are associated with white matter organization, myelination, and maturation (Zhang et al., 2008; Cancelliere et al., 2013). Several studies also demonstrated correlation between FA and motor recovery (Qiu et al., 2011; Guo et al., 2016; Wen et al., 2016). Therefore, FA is a persuasive index to depict structural changes after rTMS and to elaborate the therapeutic mechanism of rTMS in stroke recovery.

Guo et al. (2016) used FA to describe cerebral structural changes after 10-HZ rTMS on stroke patients. Compared with conventional treatment group and pretreatment status, rTMS-treated patients showed better improvement and increased FA in motor-related brain regions. And the increased FA value in the ipsilesional posterior limb of the internal capsule was correlated with the improved Fugl–Meyer Assessment score. This study was enlightening. It proved the effectiveness of rTMS in treating stroke patients and demonstrated the reliability of FA in depicting stroke recovery. However, there were some limitations. First, the DTI applied in this study had relative lower magnetic field and resolution. Second, the result may be obscured by the potential effect of acupuncture as both groups received this treatment. Third, the long-term effects of rTMS treatment were not evaluated. Therefore, longitudinal study with more advanced DTI technique and without acupuncture therapy should be carried out.

Previously our group evaluated the effectiveness of rTMS on stroke rehabilitation in a 1-year longitudinal randomized trial. We found that rTMS could facilitate motor recovery of acute stroke patients, and the effect can last to 1 month, except the function improvement on upper extremities could last for 1 year (Guan et al., 2017). We also used fMRI to compare the difference of brain functional changes between real and sham rTMS group. The study showed increased functional connectivity of ipsilesional M1 with contralesional motor-related regions (Li et al., 2016). The present study aimed to use DTI *in vivo* to investigate cerebral structural reorganization induced by rTMS in acute stroke patients.

## Materials and Methods

### Subjects

The present study was a preliminary one of an ongoing trial^1^ (Identifier: NCT03163758). Clinical data and grouping details have been previously published (Li et al., 2016; Guan et al., 2017) and are only briefly described here. We recruited stroke inpatients in our hospital from January 2013 to January 2016 and carried out routine MRI on them. The subjects’ inclusion criteria were (1) first time ischemic stroke patients within one week after onset, (2) the infarction was unilateral subcortical lesion on diffusion weighted imaging (DWI) and was restricted in the middle cerebral artery (MCA) territory, (3) motor dysfunction after stroke, (4) right-handed, and (5) no epilepsy or other mental disorders. The exclusion criteria were as follows: (1) direct damage to the cerebral cortex, (2) a history of stroke or cerebral small vessel disease, (3) tendency to hemorrhage or existing brain hemorrhage, (4) epilepsy or other mental disorders, and (5) any MRI contraindications. The study was approved by the ethics committee of the hospital, and written informed consent was obtained prior to enrollment.

The patients were separated into an active rTMS group and a sham rTMS group randomly. A random number was generated by computer, and the processing method was placed into a sealed envelope. A nurse who was not involved in the clinical evaluation was responsible for issuing and registering the number. The enrolled patients were unaware of whether they were in the real or the sham rTMS group.

### RTMS

Resting motor threshold (RMT) of ipsilesional and contralesional abductor digiti minimi muscles were determined for every patient before rTMS or sham rTMS to evaluate motor function. The RMT was defined as the lowest intensity capable of eliciting at least 5 MEPs of 50 μV peak-to-peak amplitude in 10 consecutive stimulations when single-pulse TMS was delivered to the contralateral cortex (Kobayashi and Pascual-Leone, 2003). If the minimum MEP amplitude could not be detected, then it was recorded as 100%. All patients received a 10-day active or sham rTMS beginning at about 4 days after stroke onset. The rTMS were performed using a Medtronic MagPro type magnetic stimulation device (Medtronic, Minneapolis, MN, United States) and a figure-of-eight coil (MC-B70, Medtronic). The rationale and parameters of rTMS in the present study was the same with that of our previous work (Li et al., 2016). The stimulation involved 50 trains of 20 pulses with 2-s intertrain intervals each day over the ipsilesional M1 at a frequency of 5 HZ, with the stimulus intensity set at 120% of the RMT of the unaffected extremity. In the active rTMS group, coils were placed tangent to the scalp, while in the sham group, coils were placed perpendicular to the scalp. There is no difference between the two groups in standardized therapies, including antiplatelet drugs and blood circulation protection.

### Imaging Data and Analysis

MRI data including DTI and structural images were performed on a 3.0 T MRI scanner (MAGNETOM Skyra; Siemens, Erlangen, Germany) using a 20-channel phased-array head coil. DTI images were taken with a gradient echo-planar imaging sequence (EPI) sequence. The DTI scan consisted of 30 diffusion-weighted directions with a *b*-value of 1000 s/mm^2^ and one volume without diffusion weighting (i.e., b0 image). The parameters of the DTI sequence were as follows: repetition time (TR) = 7900 ms, echo time (TE) = 94 ms, slice thickness = 2.5 mm, field of view (FOV) = 240 mm × 240 mm. High-resolution T1-weighted structural images were also acquired for all participants using the following parameters: TR = 2300 ms, TE = 2.8 ms, time of inversion (TI) = 900 ms, flip angle = 8°, slice thickness = 1.0 mm, slice gap 0.5 mm, matrix size = 256 × 256, FOV = 256 mm × 256 mm. Earplugs and earphones were used to reduce scan noises, and head motion was minimized by stabilizing the head with cushions.

The DTI data were preprocessed by PANDA software (Cui et al., 2013), following these steps: skull removal, correction of eddy-current distortion, and construction of FA maps. The FA maps generated for each patient were then transformed from individual space to a standard Montreal Neurological Institute (MNI) space via spatial normalization, and resliced with a voxel size of 2 mm × 2 mm × 2mm. All FA maps were smoothed using an isotropic Gaussian filter with a full-width-at-half-maximum of 6 mm.

Statistical analysis was performed using the Resting-State fMRI Data Analysis Toolkit (Song et al., 2011). Voxel-based analysis was used to study the difference in FA. Two-sample *t*-tests were carried out to compare the FA changes between the two groups before and after active/sham rTMS. A cluster connectivity criterion of 5 mm (edge connected), spatial smoothness of 6 mm, and a threshold of *P* < 0.05 were achieved for the results of the two-sample *t*-tests. Clusters of 83 or more voxels were regarded as the regions with significant difference between the two groups, and statistically difference maps were obtained. Changes of brain regions were projected onto an anatomical template (single T1) using the tool of xjView^2^.

## Results

**FIGURE 1:**
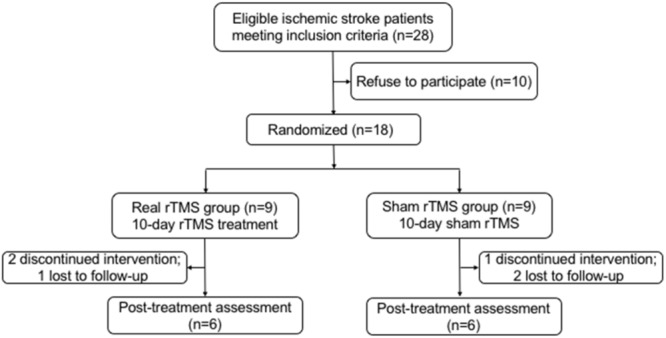
Flow chart of participants recruited in this study. rTMS, repetitive transcranial magnetic stimulation.

**FIGURE 2:**
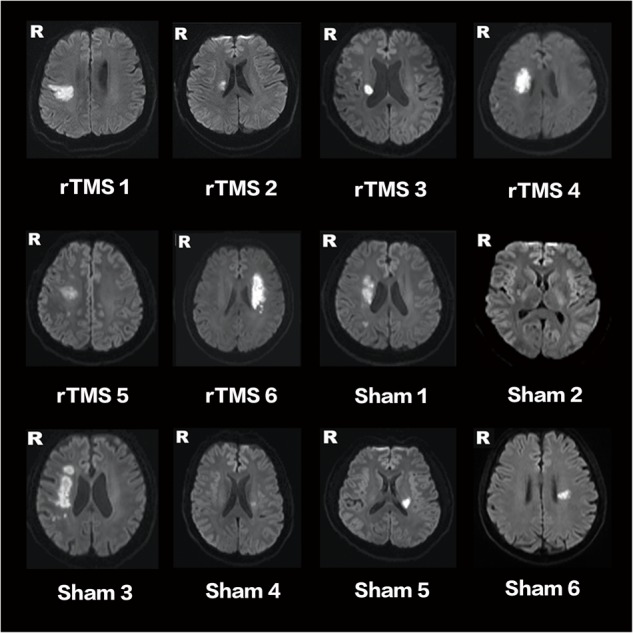
Lesion locations of stroke patients on DWI. RTMS 1–6 are patients of the rTMS treatment group. Sham 1–6 are patients of the sham group.

Of 18 participants, nine were randomly assigned to the rTMS treatment group, and the other nine were randomly assigned to the sham rTMS group. Among these 18 patients, three did not complete the intervention and three were lost during the follow-up (**Figure 1**). At last, a total of 12 patients (11 males, one female; mean age: 56.9 years old; range: 30–76 years old) with unilateral subcortical lesion in the MCA territory detected by DWI (**Figure 2**) completed the entire study. Eight of the 12 patients had right hemispheric lesion. The other four patients’ activation maps were flipped along the midsagittal plane. As a result, the affected hemisphere corresponded to the right side of the brain images for all patients. All the patients completed the 10-day rTMS/sham rTMS treatment without reporting any adverse effects.

**FIGURE 3:**
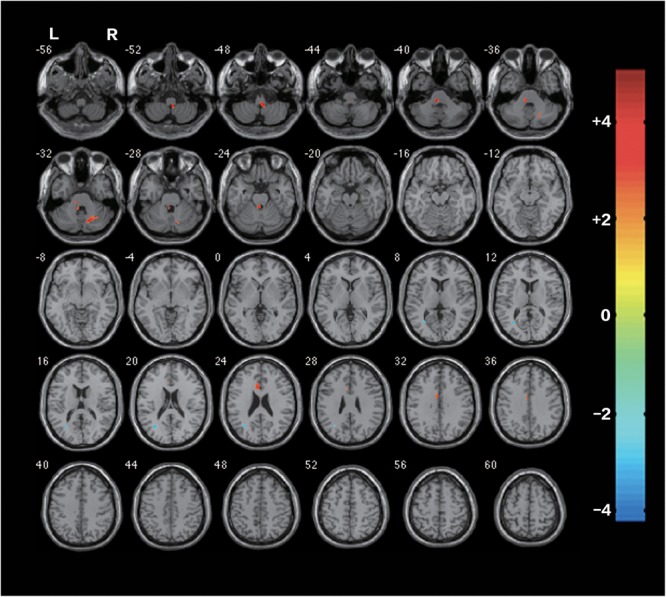
Differences of FA between the rTMS treatment group and the sham after the rTMS (two-sample *t*-test, *P* < 0.05, AlphaSim Corrected, cluster size ≥ 83 voxels). The warm-toned areas represent the regions with increased FA, while the cool-toned ones represent the regions which have decreased FA. Changes of brain regions were projected onto an anatomical template (single T1). The right side of the images refers to the ipsilesional hemisphere.

**FIGURE 4:**
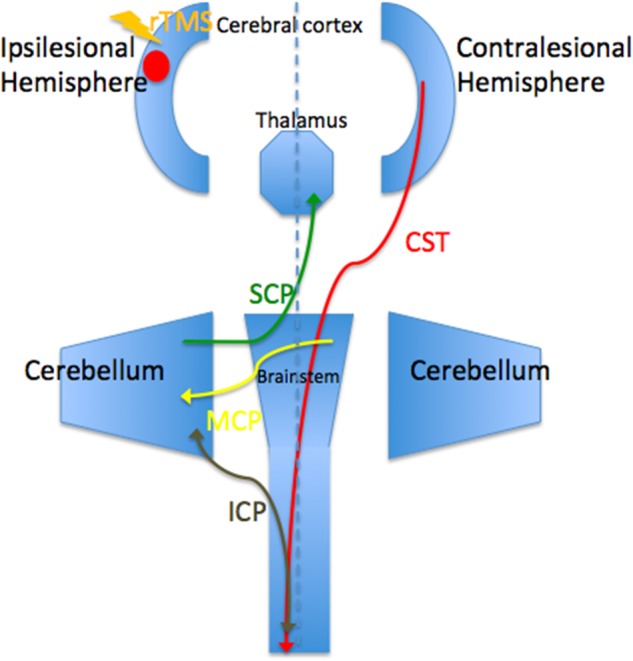
Brain regions and white matters that constitute the cortico-cerebellar loop. CST, corticospinal tract; ICP, inferior cerebellar peduncle; MCP, middle cerebellar peduncle; SCP, superior cerebellar peduncle.

Before the rTMS/sham rTMS, there is no significant difference in FA between the two groups. After the treatment, the rTMS group showed increased FA in the contralesional corticospinal tract, the pontine crossing tract, the middle cerebellar peduncle, the contralesional superior cerebellar peduncle, the contralesional medial lemniscus, and the ipsilesional inferior cerebellar peduncle (**Figure 3** and **Table 1**). These fasciculi comprise the cortex-pontine-cerebellum-cortex loop (**Figure 4**). Increased FA was also found in the body of corpus callosum and the contralesional cingulum of the treatment group compared with the sham. The contralesional posterior thalamic radiation and the contralesional posterior corona radiata showed decreased FA after the rTMS.

**Table 1:** Location for regions of significant post-rTMS changes in fractional anisotropy in **Figure 3**.

Cluster	Cluster Size (Voxels)	White Matter in the Region	Peak MNI Coordinate			Peak Intensity
			*X*	*Y*	*Z*	
Clusters of increased FA						
1	170	Pontine crossing tract; Contralesional medial lemniscus; Contralesional corticospinal tract; Ipsilesional inferior cerebellar peduncle; Contralesional superior cerebellar peduncle Con	–6	–34	–38	5.06
2	114	Middle cerebellar peduncle	20	–66	–34	4.09
3	94	Body of corpus callosum; Contralesional cingulum	–6	–2	34	3.31
Clusters of decreased FA						
4	104	Contralesional posterior thalamic radiation; Contralesional posterior corona radiata	–32	–64	14	–4.14

## Discussion

Brain reorganization after stroke is a research hotspot. However, it is challenging to delineate it *in vivo*. DTI is a non-invasive approach to display and analysis white matter (Basser et al., 1994; Xue et al., 2001). We used DTI in this study and found increased FA of the contralesional cortico-cerebellar loop and commissural fibers after excitatory rTMS. This is a preliminary result of the pre-registered clinical trial. In this trial, we first used clinical assessment scores to prove that rTMS could facilitate motor rehabilitation of stroke patients. Then we observed functional reorganization of the brain after rTMS through the method of fMRI. The present study was like the third step and focused on the structural changes after rTMS. It indicated white matter modulation in contralesional cortico-cerebellar pathways and in interhemispheric connections. The result was very enlightening and worth great attention. It may reflect contralesional compensation facilitated by the excitatory rTMS, which gives us a clue to understand the therapeutic mechanism of rTMS.

Dr. Zhang and Dr. Mori identified decreased FA in the ipsilesional cortico-cerebellar loop in stroke patients (Zhang et al., 2009). The present study carried out high frequency rTMS on stroke patients and demonstrated increased FA of the contralesional cortico-cerebellar loop. Hemispheric changes in the above studies are opposite, and the main difference between the two studies was whether rTMS was performed or not. Therefore, we can infer that excitatory rTMS-induced structural modulation of contralesional cortico-cerebellar loop. Previous studies observed significant association between cortico-cerebellar structural connectivity and motor output after stroke (Schulz et al., 2017). And cerebellar hemispheric activation after stroke had been proven to be positively related to spontaneous motor recovery (Small et al., 2002). In conclusion, we can speculate that the rTMS-induced reorganization of contralesional cortico-cerebellar fibers to compensate for the abnormality of the ipsilesional brain areas, thus facilitating motor recovery. To our knowledge, this study is the first time to show the modulation of cortico-cerebellar loop after rTMS *in vivo*, instead of through postmortem. It is important because it first prove the therapeutic effect of rTMS and second certify the potent role, which DTI plays in stroke research.

The present study also showed increased FA of the corpus callosum and the cingulum. It is possible that the cingulum may contain some fibers from the corpus callosum (Wakana et al., 2004). Corpus callosum connects homologous cortical regions of the brain (Wahl et al., 2009). Reorganization of these commissural fibers indicated strengthening communication of bilateral hemispheres. This answered the question mentioned in the introduction part. There are structural changes of the interhemispheric functionally altered brain regions after rTMS treatment. Together with our former study’s findings (Li et al., 2016), it can be inferred that the rTMS facilitates brain reorganization not only by activating the function of motor related regions but also by strengthening the fibers connecting these regions. The result is also consistent with previous study by Dr. Guo. They found increased FA value in bilateral posterior limb of internal capsule, M1 and SMA in the rTMS treatment group compared with the controls (Guo et al., 2016). Change in motor functional score was positively correlated with FA of transcallosal motor tract. Higher FA values are associated with better motor outcome (Demirtas-Tatlidede et al., 2015). Transcallosal fibers from contralesional to ipsilesional hemisphere may play a crucial role in compensating for motor dysfunction (Jang et al., 2009). Besides, the middle part of the cingulum contains motor and premotor connections (Takenobu et al., 2014). Its structural modulation after rTMS may also play a role in motor recovery. After stroke, the affected limbs are controlled by the bilateral motor cortices, and in some circumstance they are only supervised by the contralesional motor cortex without the influence of the ipsilesional one (Ago et al., 2003). Increased transcallosal integrity illustrated the crucial part that contralesional motor pathway plays in stroke recovery. Reorganization of these commissural fibers indicated the strengthening communication of the two hemispheres. Through this communication, the contralesional motor related areas could compensate better for the function of the ipsilesional one.

We only recruited ischemic stroke patients in the acute phase, which is not only innovative but also effective. Most motor rehabilitation trials enrolled chronic stroke patients, while only a few were carried out in the acute stage (Stinear et al., 2013). Thus, the effectiveness of motor rehabilitative interventions during the first few weeks after stroke onset is still largely unknown (Carey et al., 2017). Our study applied the excitatory rTMS to acute stroke patients to figure out this issue. Besides, delivering rehabilitation treatment in the acute phase would achieve better effect than in the chronic phase. Because there is spontaneous recovery in the first few weeks after stroke, and rTMS at the early stage could maximize the interaction between rehabilitation therapy and spontaneous recovery (Jørgensen et al., 1995; Cramer, 2008).

There were some limitations in the present study. First, a very limited number of patients were included in each group. Second, we only collected MRI data before and 1 month after rTMS and could not evaluate the brain structural changes in the long-term. The reasons for the limitations were that the patients’ inclusion criteria were relatively strict and that most stroke patients could not adhere to finish the multi-modal MRI scan at the acute stage. Despite these limitations, our study provided preliminary evidence in support of the therapeutic mechanism of rTMS for stroke patients. Further investigation is warranted to replicate these results and to describe how long these structural changes induced by rTMS could last.

## Ethics Statement

This study was carried out in accordance with the recommendations of the Ethics Committee of Peking Union Medical College Hospital with written informed consent from all subjects. All subjects gave written informed consent in accordance with the Declaration of Helsinki. The protocol was approved by the Ethics Committee of Peking Union Medical College Hospital (Approval No. S-067).

## Author Contributions

WZ and YG have contributed to the design of this study. JLi, YG, XZ, JLu, and XS conducted the research. JLi, ZZ, RX, and YF analyzed experimental results. JLi and WZ wrote the manuscript.

## Conflict of Interest Statement

The authors declare that the research was conducted in the absence of any commercial or financial relationships that could be construed as a potential conflict of interest.

## Footnotes

1 http://register.clinicaltrials.gov

2 http://www.alivelearn.net/xjview/
